# Evaluation of bivalirudin-based anticoagulation for extracorporeal membrane oxygenation, including patients with COVID-19

**DOI:** 10.1051/ject/2025045

**Published:** 2026-03-13

**Authors:** Miranda Raguindin, Casey Bardsley, Chelsea Bast, David Sugrue, Hasan Kazmi, Ashley Milkovits

**Affiliations:** 1 Carilion Roanoke Memorial Hospital Roanoke VA 24014 USA; 2 Hartford Hospital Hartford CT 06106 USA; 3 North Florida Regional Medical Center Gainesville FL 32605 USA; 4 Sentara Virginia Beach General Hospital Virginia Beach VA 23454 USA; 5 Duke Raleigh Hospital Raleigh NC 27609 USA

**Keywords:** ECMO (extracorporeal membrane oxygenation), Bivalirudin, Pharmacology, Acute respiratory distress syndrome (ARDS), COVID-19

## Abstract

*Background*: Although unfractionated heparin (UFH) has traditionally been used for anticoagulation during extracorporeal membrane oxygenation (ECMO), bivalirudin may be preferred due to fewer complications. A prior medication use evaluation of ECMO patients who received bivalirudin resulted in dosing updates for our pharmacist-directed bivalirudin protocol. This study intended to evaluate the efficacy and safety of bivalirudin for anticoagulation during ECMO support post-protocol initiation. *Methods*: This was a retrospective, single-center, pre-post study. Adult patients requiring ECMO support for at least 24 h and who received bivalirudin between January 1, 2015 to July 31, 2018 (pre-group) and May 1, 2019 to June 30, 2021 (post-group) were included. *Results*: There were 38 patients in the pre- and 35 patients in the post-group. The primary outcome, median time to two consecutive activated partial thromboplastin times (aPTTs) within therapeutic range for the initial goal range, was 8.9 h in the pre- and 14.2 h in the post-group (*p* = 0.517). In a subgroup analysis of the post-group, the primary outcome was higher in patients with COVID-19 (26.5 vs. 8.6 h, *p* = 0.018). The median number of dose adjustments to achieve goal aPTT was higher in COVID-19 patients (4 vs. 2, *p* = 0.017). *Conclusion*: These results suggest that a standardized pharmacist-directed protocol for bivalirudin in ECMO achieves timely therapeutic anticoagulation levels. Patients with COVID-19 trended toward longer times to two consecutive therapeutic aPTTs. Further studies are needed to evaluate dosing strategies in patients with and without COVID-19.

## Introduction

Extracorporeal membrane oxygenation (ECMO) is a therapeutic modality that allows for temporary support in pulmonary and/or cardiac failure refractory to conventional clinical treatment [1–3]. Systemic anticoagulation is necessary due to continuous contact between the patient’s blood and the foreign surfaces of the ECMO circuit components. This interaction can trigger the coagulation cascade and lead to pump thrombi, oxygenator thrombi, or fibrin stranding, resulting in thromboembolic events [4]. Hemorrhage is the most frequent and serious complication associated with ECMO support and can stem from supratherapeutic anticoagulation, thrombocytopenia, platelet dysfunction, coagulopathy, acquired von Willebrand syndrome, or hyperfibrinolysis [2]. The balance between bleeding and thrombosis in the setting of ECMO is delicate and requires close monitoring of systemic anticoagulation.

The Extracorporeal Life Support Organization (ELSO) Anticoagulation Guideline does not recommend a preferred anticoagulant [5]. Although heparin was historically the systemic anticoagulant of choice as a rapidly reversible agent with wide availability and favorable cost, there are known complications such as heparin-induced thrombocytopenia (HIT), heparin resistance, and variable clinical responses [6]. Bivalirudin is a short-acting anticoagulant that demonstrates more predictable pharmacokinetics and greater reduction of thrombin generation as compared to unfractionated heparin (UFH) [5]. Current literature reports fewer bleeding events within the first 48 h of ECMO and a longer time within goal range with bivalirudin than heparin [7, 8]. Thus, our institution adopted bivalirudin as the primary anticoagulant for use during ECMO. There are limited published data and protocols regarding dosing, which calls for further description and study into the optimal approach [9, 10]. Furthermore, there is limited literature available describing the use of bivalirudin in patients with coronavirus disease 2019 (COVID-19) receiving ECMO support [11, 12].

A cross-sectional review of all ECMO patients who received bivalirudin as their primary anticoagulant was conducted in 2018 to evaluate safety (systemic and circuit-related thrombotic events) and efficacy at our institution, in order to standardize dosing and monitoring recommendations for this patient population. Several protocol optimizations were identified, including minimizing various initial doses and initial prescribing of aPTT goals, which resulted in changes to the pharmacist-directed direct thrombin inhibitor (DTI) protocol. Since that time, all patients initiated on ECMO at this institution who have received bivalirudin as the systemic anticoagulant have utilized standardized doses and aPTT goals based on the new ECMO-specific protocol. The results of this initial review represent the pre-group of the present study. The intent of this study was to evaluate the updated pharmacist-directed ECMO bivalirudin protocol and its efficacy and safety, as well as describe dosing patterns in patients on ECMO with COVID-19.

## Materials and methods

### Study design

This was a retrospective, single-center, pre-post study. It was undertaken as a Health Care Delivery Improvement Project, and as such was not reviewed as Human Subjects Research by the Institutional Review Board.

### Intervention groups

In the 2018 cross-sectional review, adult patients who received bivalirudin for systemic anticoagulation during ECMO were evaluated from January 1, 2015, to July 31, 2018. The current study’s pre-group was comprised of this cohort. Institutional practice during this time was for initial bivalirudin dosing and aPTT goal ranges to be set by the ordering provider, individualized for each patient. Following initiation, doses were adjusted by the pharmacist per a general DTI protocol, which was not specific to ECMO patients. The primary objectives of the initial study were to characterize the patient population receiving bivalirudin for ECMO and to describe the institutional prescribing patterns. The study identified an opportunity to standardize target aPTT ranges and bivalirudin dosing, and thus, a pharmacist-directed protocol for patients on ECMO was developed and implemented in January 2019.

**Figure 1:**
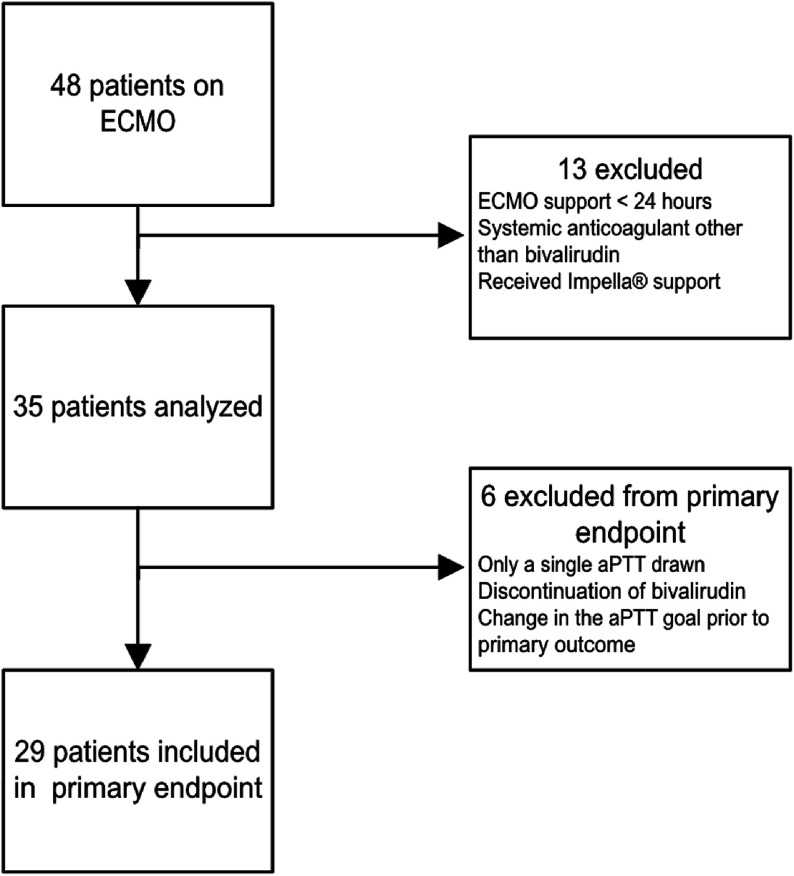
Study flow diagram for patient exclusion and primary endpoint.

Patients in the post-group were included from May 1, 2019, to June 30, 2021. Adult patients 18 years of age or older who were admitted to our institution and received ECMO support for at least 24 h were included. Patients were excluded if they received a systemic anticoagulant other than bivalirudin for ECMO anticoagulation or if they received Impella^®^ (Johnson & Johnson MedTech, Danvers, MA, USA) support (Figure 1).

### Institutional protocol

Bivalirudin for mechanical circulatory support is dosed based on a pharmacist-directed dosing protocol at our institution. The recommended initial bivalirudin rate chosen by the pharmacist is based on creatinine clearance, which is automatically calculated within the electronic health record utilizing the Cockroft-Gault equation. A low intensity (aPTT 45–60), high intensity (aPTT 60–80), or custom aPTT goal is chosen at the discretion of the cardiothoracic surgery team based on patient-specific and device-specific factors (Table 1). Once two consecutive aPTTs are within therapeutic range, aPTT monitoring may align with scheduled ECMO labs drawn every six hours instead of every two or four-hour monitoring. Two nomograms for the low and high intensity aPTT goals guide pharmacist-directed bivalirudin dose adjustments based on the aPTT (Tables 2 and 3).

**Table 1:** Institutional protocol of bivalirudin for mechanical circulatory support.

Renal Function	Initial bivalirudin infusion rate
CrCl > 60 mL/min	0.08 mg/kg/h
CrCl 30–60 mL/min	0.05–0.08 mg/kg/h
CrCl <30 mL/min or CRRT/iHD	0.03–0.05 mg/kg/h

Low Intensity (Goal aPTT 45–60)	High Intensity (Goal aPTT 60–80)
Common Uses (but not limited to): Early LVAD post-operative days (e.g., Post-op day 0–2) Higher bleeding risk VV ECMO	Common Uses (but not limited to): Later LVAD post-operative days (e.g., Post-op day 3+) Lower bleeding risk High thrombotic risk Patients with additional anticoagulation indication(s)

CrCl: Creatinine clearance; LVAD: Left ventricular assist device.

**Table 2:** Institutional protocol nomogram for low-dose bivalirudin protocol.

MCS low intensity dosing nomogram				
aPTT (seconds)	Dosage adjustments		Repeat aPTT	
	Hold infusion	Drip change	CrCl > 30 mL/min	CrCl < 30 mL/min or CRRT/iHD
aPTT < 45	n/a	Increase infusion rate by 20%	aPTT in 2 h	aPTT in 4 h
aPTT 45–60	n/a	No change (therapeutic)	aPTT in 2 h	aPTT in 4 h
aPTT 60.1–85	Hold infusion for 30 min	Decrease infusion rate by 20%	aPTT in 2 h	aPTT in 4 h
aPTT 85.1–100	Hold infusion for 30 min	Decrease infusion rate by 30%	aPTT in 2 h after resuming	aPTT in 4 h after resuming
aPTT 100.1–179.9	Hold infusion for 60 min	Decrease infusion rate by 40%	STAT aPTT every hour until aPTT < 100, then aPTT in 2 h after resuming	STAT aPTT every hour until aPTT < 100, then aPTT in 4 h after resuming
aPTT > 180	Recheck STAT aPTT as soon as infusion held to make sure the value is not an artifact due to bivalirudin contamination of specimen. After obtaining follow-up aPTT, adjust via nomogram if applicable. If aPTT remains > 180, contact CT Surgery provider to discuss recommendations			

CRRT: Continuous renal replacement therapy; iHD: Intermittent hemodialysis.

**Table 3:** Institutional protocol nomogram for high-dose bivalirudin protocol.

MCS High Intensity Dosing Nomogram				
aPTT (seconds)	Dosage Adjustments		Repeat aPTT	
	Hold infusion	Drip change	CrCl > 30 mL/min	CrCl < 30 mL/min or CRRT/iHD
aPTT < 45	n/a	Increase infusion rate by 40%	aPTT in 2 h	aPTT in 4 h
aPTT 45–59.9	n/a	Increase infusion rate by 20%	aPTT in 2 h	aPTT in 4 h
aPTT 60–80	n/a	No Change (therapeutic)	aPTT in 2 h	aPTT in 4 h
aPTT 80.1–100	Hold infusion for 30 mins	Decrease infusion rate by 30%	aPTT in 2 h after resuming	aPTT in 4 h after resuming
aPTT 100.1–179.9	Hold infusion for 60 mins	Decrease infusion rate by 40%	STAT aPTT every hour until aPTT < 100, then aPTT in 2 h after resuming	STAT aPTT every hour until aPTT < 100, then aPTT in 4 h after resuming
aPTT >180	Recheck STAT aPTT as soon as infusion held to make sure the value is not an artifact due to bivalirudin contamination of specimen. After obtaining follow-up aPTT, adjust via nomogram if applicable. If aPTT remains > 180, contact CT Surgery provider to discuss recommendations			

### Study outcomes and data collection

Patient baseline and ECMO characteristics were collected. Baseline coagulopathy was defined as an international normalized ratio (INR) greater than 1.5 or platelets less than 50 K/µL. Incidence of renal failure during ECMO was defined as initiation of continuous renal replacement therapy (CRRT) or acute kidney injury as defined by the KDIGO guidelines [13]. For our non-ECMO bivalirudin protocol, aPTT monitoring decreases to once daily once two consecutive aPTTs are within the goal range. Therefore, the primary outcome of this study was time to two consecutive aPTTs within the therapeutic range for the initial goal range.

Safety outcomes of bleeding and venous thromboembolism (VTE) were evaluated. Bleeding was defined as a decrease in hemoglobin of at least 3 g/dL based on the definition from the pre-group (which was previously collected data) to allow for statistical comparison. Major bleeding based on the ELSO guideline, defined as a hemoglobin drop of at least 2 g/dL or transfusion of at least 10 mL/kg of packed red blood cells (PRBCs) in 24 h, was collected in the post-group only for further characterization of clinical significance [5]. Systemic VTE was defined as a newly diagnosed deep vein thrombosis, pulmonary embolism, thrombotic stroke, or intracardiac thrombus. Incidence of circuit thrombosis was assessed using twice-daily ECMO nurse specialist notes.

Secondary endpoints evaluated in the post-group included percent of aPTTs out of range after achieving two consecutive aPTTs within therapeutic range in the post group; percentage of supratherapeutic and subtherapeutic aPTT values; number of bivalirudin dose adjustments to achieve two consecutive therapeutic aPTTs; and number of bivalirudin aPTT goal adjustments. A subgroup analysis of COVID-19 patients was planned for the post-group.

### Statistical analysis

Student’s T-test or Mann-Whitney U Test was used to compare continuous variables. Medians and interquartile ranges were used in reporting non-parametric data. Categorical variables were compared using the Chi-square or Fisher’s exact test. Statistical analysis was completed using SAS software, Version 9.4M7.

## Results

### Baseline characteristics

There were 38 patients in the pre-group and 35 patients in the post-group. One patient in the post-group was included twice due to two separate ECMO periods. Baseline patient characteristics between groups were similar (Table 4). Forty percent of patients in the post-group were receiving therapeutic anticoagulation prior to ECMO initiation.

**Table 4:** Baseline characteristics.

	Pre-Group (*N* = 38)	Post-Group (*N* = 35)	*P*-Value
Age, years, median (IQR)	63.5 (57–70)	55 (44.5–65.6)	0.181
Sex, female, *n* (%)	10 (26.3%)	9 (25.7%)	0.953
Weight, kg, median (IQR)	97.1 (84.9–109.3)	96.2 (85.3–107.1)	0.395
BMI, kg/m^2^, median (IQR)	30 (26–34)	33.1 (28.8–37.5)	0.277
Baseline coagulopathy, *n* (%)	16 (42.1%)	16 (45.7%)	0.756
Therapeutic anticoagulation prior to ECMO, *n* (%)	N/A	14 (40%)	N/A
VTE		7	
ACS		5	
AFib		1	
HIT		1	

BMI: Body mass index; VTE: Venous thromboembolism; ACS: Acute coronary syndrome; AFib: Atrial fibrillation; HIT: Heparin-induced thrombocytopenia.

While the pre-group was predominantly cannulated for veno-arterial (VA) ECMO, the post-group was treated equally with both VA and veno-venous (VV) ECMO, with the post-group also having predominantly peripheral cannulation. The median duration of ECMO for both groups was about one week. Respiratory illness was a more common indication for ECMO in the post-group, with 25.7% of all patients having COVID-19. Incidence of renal failure during ECMO was significantly lower in the pre-group (54.3% vs. 74.3%, *p* = 0.033; Table 5).

**Table 5:** ECMO characteristics.

	Pre-Group (*N* = 38)	Post-Group (*N* = 35)	P-Value
Type, *n* (%)			<0.001
VA	36 (94.7%)	19 (54.3%)	
VV	2 (5.3%)	16 (45.7%)	
Cannulation site, *n* (%)			0.005
Central	17 (44.7%)	3 (8.6%)	
Peripheral	21 (55.3%)	32 (91.4%)	
Days on ECMO, median (IQR)	6.9 (3.1–10.7)	7.1 (0–17)	0.163
Indication, *n* (%)			N/A
Respiratory illness	5 (13.2%)	15 (42.9%)	
COVID-19	0 (0.0%)	9 (25.7%)	
Cardiogenic shock	11 (28.9%)	3 (8.6%)	
Intra-procedural or intra-operative MCS	10 (26.3%)	8 (22.9%)	
Cardiac arrest	7 (18.4%)	4 (11.4%)	
Procedural or operative cardiac arrest	5 (13.2%)	5 (14.3%)	
Renal failure on ECMO, *n* (%)	19 (54.3%)	26 (74.3%)	0.033

VA: Veno-arterial; VV: Veno-venous.

### Primary outcome

The median time to two consecutive aPTTs within therapeutic range for the initial goal range was 8.9 h in the pre-group and 14.2 h in the post-group (*p* = 0.517; Table 6). Excluding the nine COVID-19 patients from the post-group analysis, the median time was 8.6 h (*p* = 0.615). Four patients in the pre-group and six patients in the post-group were not included in the analysis of the primary outcome due to only having a single aPTT drawn in the initial goal range, discontinuation of bivalirudin, or a change in the aPTT goal prior to two consecutive therapeutic aPTTs.

**Table 6:** Results.

	Pre-Group (*N* = 38)	Post-Group (*N* = 35)	Post-Group excluding COVID-19 patients (*N* = 26)	*P*-Value
Time to two consecutive aPTTs within therapeutic range for the initial goal range, hours, median (IQR)	8.9 (2.0–15.8)	14.2 (4.3–24.1)	N/A	0.517
		N/A	8.6 (3.2–14.1)	0.615
Percent aPTTs out of range after 2 consecutive aPTTs within documented therapeutic aPTT goal, median (IQR)	N/A	14.3 (3.35–25.3)	9.1 (0–20.1)	N/A
Percent supratherapeutic aPTT values, median (IQR)	N/A	4.7 (0–14.6)	N/A	N/A
Percent subtherapeutic aPTT values, median (IQR)	N/A	8.3 (0–19.4)	N/A	N/A
Number of bivalirudin aPTT goal adjustments, median (IQR)	N/A	1 (0–2)	N/A	N/A
Renal Failure, median (IQR)		2 (0–3)		
Without renal failure, median (IQR)		1 (0–6)		

### Secondary outcomes (post-group)

The post-group had further aPTT characterization (Table 6). The median percentage of aPTTs out of range after achieving two consecutive therapeutic aPTTs was 14.3% including all patients and 9.1% excluding COVID-19 patients. The median percentage of subtherapeutic aPTT values was higher at 8.3% compared to 4.7% supratherapeutic values. There was a median of one aPTT goal range adjustment while patients were on bivalirudin.

Twelve patients died while on ECMO in the post-group, three of whom were COVID-19 patients. Seventeen patients, including two COVID-19 patients, survived to hospital discharge.

### Safety outcomes

Safety outcomes of bleeding and VTE are shown in Table 7. Bleeding occurred in 23.7% of the pre-group compared to 11.4% in the post-group (*p* = 0.172). Additional endpoints related to bleeding were collected for the post-group only. A major bleed occurred in 31.4% of patients in the post-group. The most common type of major bleed was retroperitoneal.

**Table 7:** Adverse events.

	Pre-Group (*N* = 38)	Post-Group (*N* = 35)	*P*-Value
Hgb Drop ≥ 3 g/dL, *n* (%)	9 (23.7%)	4 (11.4%)	0.172
Major Bleed, *n* (%)	N/A	11 (31.4%)	N/A
Hgb Drop ≥ 2 g/dL		11 (31.4%)	
Transfusion of at least 10 mL/kg of PRBCs in 24 h		5 (14.3%)	
Type of Major Bleed, *n* (%)	N/A		N/A
Retroperitoneal		4 (36.4%)	
Pulmonary		1 (9.1%)	
CNS		0	
Requires Surgical Intervention		2 (18.2%)	
Other		4 (36.4%)	
Systemic VTE, *n* (%)	3 (7.9%)	2 (5.7%)	0.763
DVT	1	1	
PE	0	0	
CNS	1	0	
Intracardiac	1	1	
Circuit thrombosis, *n* (%)	34 (89.5%)	24 (68.6%)	0.027
Oxygenator	34	23	
Hemofilter	0	0	
AV Bridges/Cannulas	0	1	

Hgb: Hemoglobin; PRBCs: Packed red blood cells; CNS: Central nervous system; DVT: Deep vein thrombosis; PE: Pulmonary embolism; AV: Arterio-venous.

Systemic VTE occurred in 7.9% of the pre-group and 5.7% of the post-group (*p* = 0.763). There was significantly more circuit thrombosis in the pre-group (89.5% vs. 68.6%, *p* = 0.027). The most common site of circuit thrombosis was the oxygenator.

### COVID-19 subgroup analysis

Additional analysis was completed for select endpoints in the post-group comparing the nine COVID-19 patients to the 26 patients without COVID-19 (Table 8). Renal failure was significantly more common in the COVID-19 subgroup, with all patients developing renal failure during ECMO compared to 65.4% of those without COVID-19. The time to two consecutive aPTTs within the goal range was significantly longer in patients with COVID-19 by 18 h (26.5 vs. 8.6 h, *p* = 0.018), although the median bivalirudin dose at which the therapeutic aPTT was achieved was not significantly different between groups. Additionally, there was a wide range of therapeutic bivalirudin doses for COVID-19 patients (0.025–0.2 mg/kg/h).

**Table 8:** Post-group COVID-19 subgroup analysis.

	COVID-19 (*n* = 9)	Without COVID-19 (*n* = 26)	*P*-Value
Renal failure, *n* (%)	9 (100%)	17 (65.4%)	0.046
Time to 2 consecutive aPTTs within goal range, hours, median (IQR)	26.5 (17.7–37.9)	8.6 (3.2–14.1)	0.018
Initial dose, mg/kg/h, median (IQR)	0.05 (0.05–0.06)	0.055 (0.05–0.08)	0.426
Dose at which therapeutic aPTT achieved, mg/kg/h, median (IQR)	0.055 (0.031–0.137)	0.048 (0.032–0.086)	0.558
Number of dose adjustments until goal aPTT achieved, median (IQR)	4 (2–6)	1 (0–2)	0.017
Number of aPTT goal adjustments, median (IQR)	1 (0–1)	1 (0–1)	0.968

## Discussion

With the growing use of bivalirudin for ECMO anticoagulation and the subsequent implementation of a pharmacist-directed ECMO-specific bivalirudin protocol at our institution, this study found that the median time to two consecutive aPTTs within goal range was 8.9 h. This was not statistically different from the time of 14.2 h prior to the protocol implementation, although both studies had small sample sizes of less than 40 patients. Patients with renal failure required a median of two dose adjustments, while patients without renal failure required a median of one adjustment. Although the differences between groups were not statistically significant, a median time of around 9 h to achieve a therapeutic aPTT is clinically appropriate, and we concluded that the starting dose ranges in our current protocol are reasonable. It took less than 14.1 h to achieve our primary endpoint of time to two consecutive therapeutic aPTTs in 75% of the patients, and only three patients without COVID-19 took greater than 24 h to achieve our primary endpoint. These findings are consistent with those of Netley and colleagues, who reported in a retrospective review of their dosing protocol that all 11 patients were within the aPTT target range within 24 h [10].

Patients in the post-group, excluding those with COVID-19, had a low rate of 9.1% of aPTTs out of range after two initial consecutive therapeutic aPTTs. This demonstrated high consistency of aPTTs maintained within goal range and suggests that subsequent monitoring can be reduced to once daily to decrease unnecessary use of resources, including personnel time and physical supplies from phlebotomy, pharmacy, and the laboratory, due to the collection of fewer aPTTs. Rates of aPTTs out of range were reported at 14.3% by Kaseer and colleagues and 33.7% by Netley and colleagues, although our definition differed in that we reported the consistency of aPTTs maintained within goal range once it was initially achieved [8, 10]. Ten patients were excluded from the primary analysis due to not meeting the endpoint of two consecutive therapeutic aPTTs within the initial goal range; while this may have influenced the findings, it also reflects the clinical complexity of ECMO patients. Therapeutic bivalirudin doses in this study were similar to those used by Ranucci and colleagues, although, as noted by Sanfilippo and colleagues, differences in anticoagulation monitoring and goal ranges in studies make direct comparisons in therapeutic bivalirudin rates difficult [7, 9]. Mortality on ECMO and survival to hospital discharge were similar to rates seen with other studies [7, 8].

About one-third of patients in the post-group had a major bleed. However, this was mostly due to a decrease in hemoglobin of 2 g/dL or more as compared to requiring transfusion of PRBCs, and only two patients required surgical intervention, possibly indicating a lower bleed severity. Major bleeding rates in the post-group in this study were similar to those found by Kaseer and colleagues when considering the definition included a hemoglobin drop of at least 3 g/dL [8]. While systemic VTE occurrence was low and not different between groups, there was over 20% less circuit thrombosis in the post-group, despite a significant portion having COVID-19, which has previously been documented as a pro-thrombotic disease [11]. When comparing aPTT goals, the pre-group had lower aPTT goal ranges, which may account for the lower incidence of circuit thrombosis in the post-group. Additionally, 40% of patients in the post-group were already receiving full anticoagulation prior to ECMO, which may have contributed to a reduction in circuit thrombosis in the post-group as well. Circuit thrombosis rates, although lower in our post-group than pre-group, remained higher than those reported in other studies [6, 8]. The higher rate may be due to differing definitions, as one study defined circuit thrombosis as a thrombus requiring a change in ECMO cannula, tubing, pump, and/or oxygenator. Of note, only five patients in the post-group required oxygenator change. Our assessment of circuit thrombus was also based on reports of visual thrombus from nursing notes, an endpoint which was consistent with the previously collected data from the pre-group.

### COVID-19 subgroup analysis

The differences in baseline characteristics between groups, including VV ECMO, pre-ECMO therapeutic anticoagulation, respiratory illness, and renal failure, are likely due to the COVID-19 population in the post-group. Hypercoagulability has been well documented in COVID-19 patients. It has been proposed that the inflammatory response from cytokine release interferes with endothelial function and activates the coagulation cascade [12, 14]. This could help explain why 20% of patients in the post-group were receiving anticoagulation prior to ECMO for VTE. COVID-19 patients in our study required a wider range of bivalirudin doses, had a higher percentage of aPTTs out of range, and took longer to reach therapeutic aPTTs. All patients in the COVID-19 group had renal failure, but they required similar bivalirudin doses to those without renal failure. Since the starting doses in our protocol are lower for renal impairment, it took longer to become therapeutic due to the need for multiple dose adjustments in the COVID-19 group. Prevalence of renal failure requiring renal replacement therapy has been reported in patients with COVID-19, with proposed mechanisms including cytokine release leading to inflammation and thrombotic microangiopathy [14]. In our patients, CRRT was typically started for renal failure and not volume management or cytokine release alone. Given the unique pathophysiology of COVID-19 and bivalirudin renal dose findings not in line with the rest of the cohort without COVID-19, no changes were made to our institutional protocol.

Although the range of therapeutic doses is not well documented in the literature, the reported average doses of 0.18 and 0.2 mg/kg/h fall within the range of our study [11, 12]. Interestingly, the percentage of aPTTs out of range does not necessarily correlate with other published retrospective findings of bivalirudin use in COVID-19 patients on ECMO. One study found that the percentage of aPTTs at goal was maintained more consistently in patients with COVID-19 versus non-COVID-19. However, only 11% of their COVID-19 patients required renal replacement therapy after ECMO initiation compared to 26% non-COVID-19 patients, both rates which are much lower than in our current study [11]. Bissell and colleagues reported a median time to therapeutic range of 20 h, in line with the findings of this study, despite only 55% of their patients being on CRRT compared to 100% of patients in our study [12]. However, these two studies included 42 and 33 COVID-19 patients, respectively, compared to the sample size of nine patients at our institution, and only utilized a single aPTT goal range of 60–80 s [11, 12].

### Limitations

This study has limitations. It was retrospective in nature, thereby relying on accurate documentation in the electronic medical record. Previously collected data was used for the pre-group, and no data was recollected. Some endpoints were defined in a non-standard way to allow direct comparison between the post-group and the previously collected data in the pre-group, while other endpoints were not previously collected in the pre-group to allow for comparison in the post-group. This study did not seek to evaluate the effectiveness of nomogram dose adjustments for achieving therapeutic aPTTs, and thus, not all aspects of the protocol could be assessed for appropriateness. There was a small sample size in both groups, especially when evaluating the COVID-19 subgroup, and findings for that population should only be considered hypothesis-generating. The primary outcome was also not able to be evaluated for all patients in an already small group. The time to first circuit thrombosis was not collected, which may have allowed us to draw more definitive conclusions on pre-ECMO systemic anticoagulation.

## Conclusion

In conclusion, the results of this study suggest that adopting a standardized pharmacist-directed protocol for bivalirudin in ECMO rapidly achieves therapeutic anticoagulation levels. Furthermore, the recommended bivalirudin starting doses in our current institutional protocol are appropriate. Based on the data in the post-group, our institution’s current DTI protocol monitoring was changed to once daily aPTTs after achieving two consecutive therapeutic aPTTs. Given the current published evidence, further studies evaluating bivalirudin dosing strategies and appropriate aPTT goals in both patients with and without COVID-19 are needed to better evaluate efficacy and safety outcomes and to further elucidate dosing considerations.

## Data Availability

The research data associated with this article are included in the article.
